# P05-16 Does the effect of steps per hour on ambulatory blood pressure differ between work hours and leisure time? A cross-sectional study among cleaners in Denmark

**DOI:** 10.1093/eurpub/ckac095.083

**Published:** 2022-08-29

**Authors:** Vivian Rueskov Poulsen, Mathilde Baumann, Ole Steen Mortensen, Korshøj Mette

**Affiliations:** 1 Department of Occupational and Social Medicine, Holbæk Hospital, Holbæk, Denmark; 2 Section of Social Medicine, Department of Public Health, University of Copenhagen, Copenhagen, Denmark

**Keywords:** blood pressure, physical activity, steps per hour, resting heart rate, health paradox

## Abstract

**Objective:**

The physical activity health paradox, describing contrasting long-term effects of domain-specific physical activity on health, states occupational physical activity (OPA) to be hazardous and leisure time physical activity (LTPA) to be beneficial for health. Yet, the acute effects of OPA and LTPA on cardiovascular risk factors are sparsely investigated. The aim of this study was to investigate the acute effects on ambulatory blood pressure (ABP) from steps per hour during work and leisure time among cleaners in Denmark.

**Methods:**

Data were obtained from a cluster randomized worksite intervention among 91 cleaners in Denmark. Data included a questionnaire, objective physical measurements of weight, height, BMI, ABP and steps per hour. The latter was measured during work and leisure time within a maximum of four continuous days. A preliminary linear regression analysis was conducted as a mixed model including random intercept and slope, allowing for both within- and between-participant variability. The analysis was adjusted for sex, age, job seniority, medication use, smoking, self-reported fitness and BMI. Changes in ABP (mmHg) were estimated per 100 steps/hour.

**Results:**

Mean steps/hour were 1333.4 (*SD* ± 404.1) during work and 530.8 (*SD* ± 234.1) during leisure time. The resting ABP was systolic 122.4 mmHg (*SD* ± 20.5) and diastolic 80.7 mmHg (*SD* ± 12.8). In total, 15.4% of the population were hypertensive (≥140/90 mmHg, or using anti-hypertensives). The ABP did not seem to differ from exposure to steps taken during work (systolic -0.44 mmHg, 95% CI: -1.07-0.20, diastolic 0.04 mmHg, 95% CI, -0.43-0.36) and leisure (systolic -0.42 mmHg, 95% CI, -1.54-0.70, diastolic 0.30 mmHg, 95% CI, -0.49-1.08). Either did the amount of steps taken seem to affect the ABP during work or leisure.

**Conclusion:**

Our findings show no significant association between steps per hour and ABP, and no contrasting effects between work and leisure time. These acute mechanisms fostering the divergent results need to be further investigated to improve the understanding of the physical activity health paradox.

